# A Collection of Designed Peptides to Target SARS-CoV-2 Spike RBD—ACE2 Interaction

**DOI:** 10.3390/ijms222111627

**Published:** 2021-10-27

**Authors:** Narcis Fernandez-Fuentes, Ruben Molina, Baldo Oliva

**Affiliations:** 1 Institute of Biological, Environmental and Rural Science, Aberystwyth University, Aberystwyth SY233EB, UK; 2 Structural Bioinformatics Lab, Department of Experimental and Health Sciences, University Pompeu Fabra, 08003 Barcelona, Spain; ruben.molina-fernandez@upf.edu

**Keywords:** peptide design, SARS-CoV-2, ACE2, protein–protein interactions, databases

## Abstract

The angiotensin-converting enzyme 2 (ACE2) is the receptor used by SARS-CoV and SARS-CoV-2 coronaviruses to attach to cells via the receptor-binding domain (RBD) of their viral spike protein. Since the start of the COVID-19 pandemic, several structures of protein complexes involving ACE2 and RBD as well as monoclonal antibodies and nanobodies have become available. We have leveraged the structural data to design peptides to target the interaction between the RBD of SARS-CoV-2 and ACE2 and SARS-CoV and ACE2, as contrasting exemplar, as well as the dimerization surface of ACE2 monomers. The peptides were modelled using our original method: PiPreD that uses native elements of the interaction between the targeted protein and cognate partner(s) that are subsequently included in the designed peptides. These peptides recapitulate stretches of residues present in the native interface plus novel and highly diverse conformations surrogating key interactions at the interface. To facilitate the access to this information we have created a freely available and dedicated web-based repository, Pep*I*-Covid19 database, providing convenient access to this wealth of information to the scientific community with the view of maximizing its potential impact in the development of novel therapeutic and diagnostic agents.

## 1. Introduction

In December 2019, a disease affecting predominantly the respiratory system emerged caused by a new coronavirus, SARS-CoV-2, on what has become the COVID-19 pandemic. The SARS-CoV-2 virus is closely related to SARS-CoV responsible for an outbreak in 2002 [1]. To infect the cells, both SARS-CoV-2 and SARS-CoV use the angiotensin-converting enzyme 2 (ACE2) as a keyhole, binding to it via the receptor-binding domain (RBD) of the spike protein together with the serine protease TMPRSS2 [2,3,4,5]. This interaction is a key step in viral infection of the cells and thus, preventing this association arises as a valid therapeutic strategy. Indeed, the targeting of RBD has been highlighted in a number of recent works ranging for the use of soluble human ACE2, i.e., not anchored to the cellular membrane, able to prevent virus infection in organoids [6], engineered variants of soluble human ACE2 showing higher affinity to RBD to be used as decoys [7], computationally designed mini-proteins to mimic ACE2 with outstanding pico-molar binding affinities [8], and a range of monoclonal antibodies [9,10], nanobodies [11,12], as well as aptamers [13,14] that target RBD.

Peptides can be also used as decoys to target protein-protein interactions (PPIs). The use of peptides to modulate PPIs have been gaining traction in recent years given the limitation of traditional small, drug-like, chemicals to target interfaces [15]. In the context of viral infection examples such as the FDA-approved peptide Enfurvirtide [16] and further research (reviewed in [17,18]) illustrate the use of peptides as potential agents to block PPIs. In fact, a number of recent publications has shown promising results on the use of peptides to blocks the entrance of SARS-CoV-2 virus using peptides derived from native element of the interaction humanACE2 and SARS-CoV-2 spike [19,20,21] including designed peptides targeting of the heptad repeat 1 region [22], the RBD domain [23,24,25], with validation in cell-cultures [26,27] and tissues [28]. While these approaches rely on the sequence diversification of existing native elements, ours complements these efforts by providing novel sequences (next).

The work presented here is framed within the deluge of structural information available on SARS-CoV-2 proteins and human-viral complexes, which has grown dramatically since the start of the pandemic. The structure of the interaction between ACE2 and the RBD of the spike protein, both for the SARS-CoV [2] and SARS-CoV-2 [4,29] viruses are known, and have been also solved bound to a number of monoclonal antibodies [9,10] and nanobodies [9,10]. We have used this information to guide the computational modelling and design of orthosteric peptides to target the RBD using PiPreD [30]. We have therefore leveraged on the existing structural information available on the interaction between RBD and ACE2 to model and design peptides targeting two relevant interactions for the complex: (1) the interaction between RBD and ACE2 both SARS-CoV and SARS-CoV2; and (2) the interaction between ACE2 monomers.

The outcome of our research is a large and highly diverse set of peptides designed specifically to target the surface described above. The designed peptides not only recapitulate native elements of the interaction but also provide additional conformations that also preserve the key elements of the interactions. The clustering the resulting peptides by structure identify preferred conformations in specific regions of the surface together with sequence profiles associated to them. We have compiled this information in a public repository: Pep*I*-Covid19 to make it available to the scientific community, particularly experimental scientists researching on novel therapeutic and diagnostic agents. In addition to the sequences included in repository, the modelled structures of the protein–peptides complexes can represent also the starting point for further refinement and redesign using alternative approaches that can in turn yield novel peptide sequences. Finally, we aim at keeping Pep*I*-Covid19 an up-to-date and alive resource, and thus any new structural data on protein complexes related to COVID-19, and SARS mediated diseases at large, will be duly processed and included in the repository.

## 2. Results and Discussion

### 2.1. Interfaces Targeted by Designed Peptides

The structure of 5 protein complexes were used in the modelling and design of peptides to target RBD. Two of them include interactions with ACE2, two with monoclonal antibodies (mAb) and one with a nanobody. In addition, peptides were also derived to target the interface between ACE2 monomers (Table 1). There were therefore two different interfaces considered: the interface between the RBD domain of viral spike protein and ACE2 and the interface that mediates the interaction between ACE2 monomers. The ACE2 protein forms a homodimer that is recognized by two trimeric spike proteins mediated by the RBD domain [4] and thus both interfaces can be potentially targeted to prevent infection. As shown in Table 1, the largest area corresponded to the ACE2/ACE2 dimerization interface while the smallest was the RBD/nanobody H11 D4; also reflected in the number of anchors residues, i.e., elements used to model the peptides.

**Figure 1:**
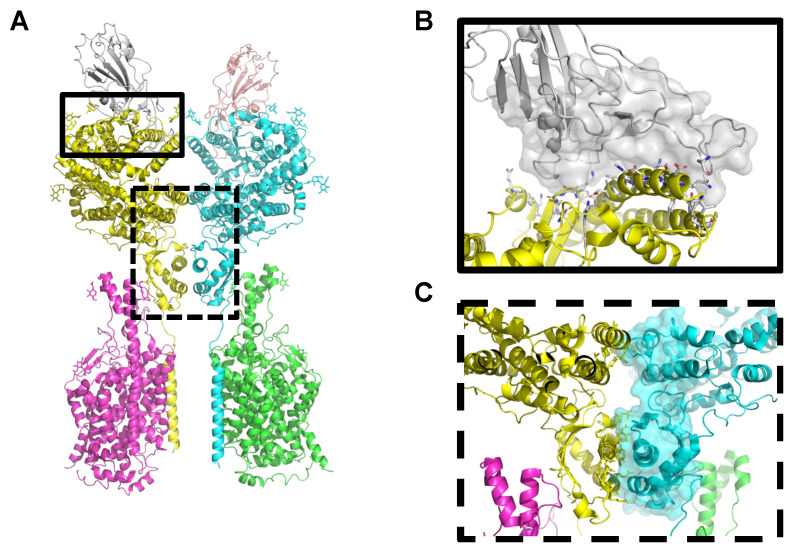
Interfaces targeted by designed peptides in the case of SARS-CoV-2 RBD and full length ACE2. (**A**) Cartoon representation of full length human ACE2 (yellow, cyan)/BoAT1 (magenta, green) and RBD (grey, pale pink). (**B**,**C**) Interface targeted by peptides where (**B**) shown and enlarged view of the interface between ACE2 (yellow, cartoon) and RBD (grey, surface) and (**C**) and enlarged view of the interface between ACE2 monomers (yellow and cyan).

The interface between RBD and ACE2 account for an area of around 2000 Ang^2^ is dominated by extensive contacts between loops of RBD and the alpha-1 helix (H1) of ACE2 (Figure 1A,B and Supplementary Figure S1 for SARS-CoV RDB/ACE2). In the case of complexes of RBD and mAbs, the area of the interface is comparable to that of the ACE2 and the dominant elements mediating the interaction with RBD, as expected, are the loops of the complementarity determining regions (CDRs) [31] (Supplementary Figures S2 and S3). Finally, nanobody H11 D4 also recognize RBD although through a smaller interface and were the dominating structural elements are also loops (Supplementary Figure S4).

The interface between ACE2 monomers is larger than RBD/ACE2 interface accounting for an area of around 2700 Ang^2^ (Figure 1, panel C, surface representation). The structural elements mediating the interactions between both monomers are mainly stacking mirroring helices from both monomers. As discussed in the publication describing the structure [4], ACE2 dimerizes through two different interfaces involving the peptidase and Neck domains. In the central region, between both interfaces, there are not direct interactions between ACE2 monomers; however, it was also considered for the modelling of peptides. Interestingly, peptides spanning the two structurally distant interfaces, include native elements of the interface but also de novo interactions (see below).

### 2.2. Designed Peptides Show Specific Sequence Preferences Depending on SARS-CoV or SARS-CoV-2 RBD/ACE2 Interface

As discussed in a previous work, the structural similarity of the RBD of both SARS-CoV and SARS-CoV2 viruses is very high but changes on interface residues have a positive contribution to the interaction with ACE2 in the case of SARS-CoV-2 by providing additional atomic interactions [32]. Indeed, while the overall structure is conserved the sequences of the RBD in both SARS-CoV and SARS-CoV-2 are less so. Given the nature of this study, including information of a closely related complex can help increasing the specificity of peptide sequences by direct comparison of both pools of designed peptides, i.e., SARS-CoV and SARS-CoV-2.

**Figure 2:**
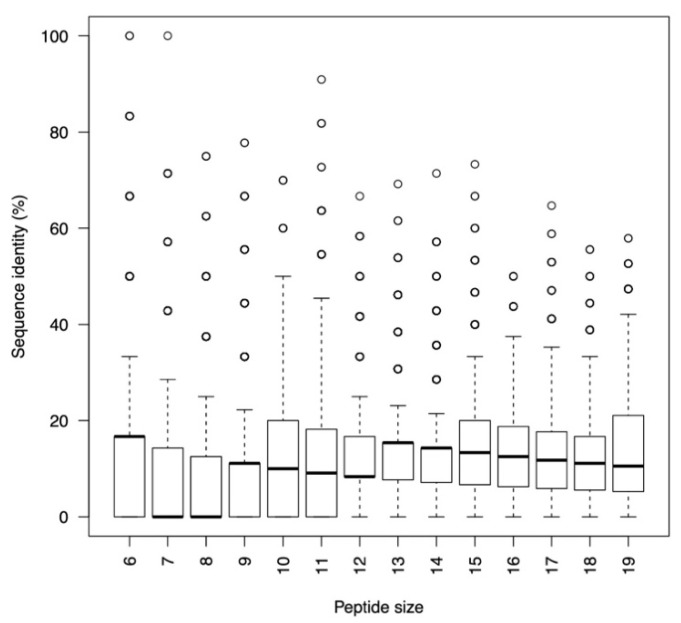
Box plot showing the distribution of sequence identity. 10,000 pairs of sequences of different sizes (*X*-axis) designed to target the RBD of SARS-CoV and SARS-CoV-2 were selected randomly and aligned.

Given the structural similarity between both RBDs, the interface targeted in the case of SARS-CoV (Supplementary Figure S1) has similar features to that of SARS-CoV-2 (Figure 1). This fact is reflected not only on a comparable area of their respective interfaces but also in the number on interface or anchor residues. The sequences of the peptides designed to target both surfaces were however quite different. As shown in Figure 2, the distribution of sequence identity of 10,000 peptide pairs with size ranging from 6 to 19 that were chosen randomly was very low. Even in the case of short peptides and besides outliers, the average sequence identity of any given pair was below 20% decreasing as the size of peptides increase. This is an important observation that show that even though both RBD in SARS-CoV and SARS-CoV-2 shared an important structural similarity and do target the same receptor, ACE2, the interface presents unique features that could be exploited by developing specific peptides.

### 2.3. Structural Diversity among Designed Peptides

**Figure 3:**
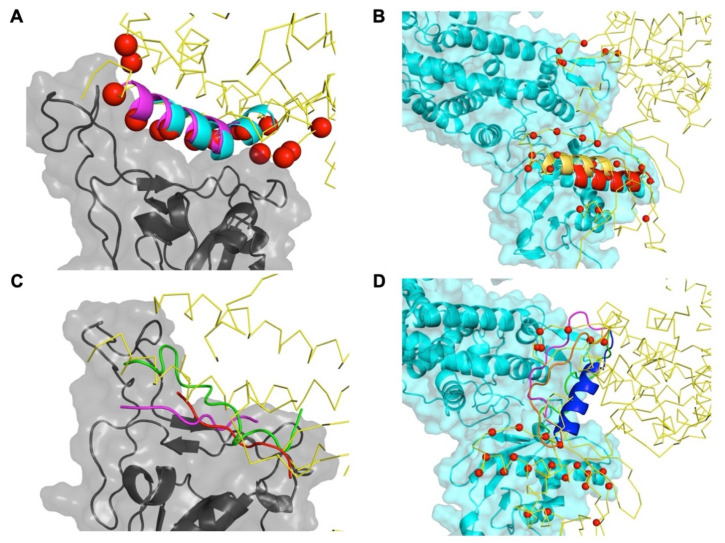
Structural model of the peptides targeting RBD and ACE2 dimerization interfaces. Panels A and C shows structural model of several peptides designed to target SARS-CoV-2 RBD in cartoon representation recapitulating native elements of the interface (**A**) or presenting novel conformations (**C**). RBD is shown in grey and cartoon and surface representation while ACE2 is shown as a trace of C-alpha (yellow); anchor residues are shown as red spheres. Panels B and D show the structural models of peptides targeting ACE2 dimerization in cartoon representation also recapitulating native elements (**B**) or new conformations (**D**). ACE2 is presented both in cartoon and surface (cyan) and as a trace of C-alpha (yellow); anchor residues are shown as red spheres.

PiPreD relies on native elements of the interface to target in the form of disembodied interface residues, or anchor residues, without considering the connectivity between them. Thus, the resulting designed peptides are not mere regions or elements of the interface as spliced from the native complex. Nonetheless, designed peptides recapitulate these native elements of the interface. In the case of RBD/ACE2 interface, different peptides recapitulated the main H1 helix (partially or entirely) with minor conformation adjustments (Figure 3A) Likewise, in the case of the ACE2/ACE2 interface, peptides also show a helical conformation similar to the helix that mediates the interaction between ACE2 monomers (Figure 3B) Redesigned native elements, such as the main H1 helix, has been shown to inhibit the interaction between RBD and ACE2 ([8,25,26,27]).

Besides recapitulation of native structural elements, designed peptides also presented novel conformations not observed in the native complexes. These peptides incorporated native elements of the interface in the form of anchor residues, mimicking or surrogating them, but presented in novel conformations as well as novel interactions to the targeted surface. For instance, in the case RBD/ACE2 interface, an interaction largely dominated by H1 helix, a large number of peptides, accounting for up to half of the designed ones, presented an extended conformation. Three examples are shown Figure 3C with peptides presenting an extended conformation mimicking the interactions of the main H1 helix. Peptides targeting the ACE2 dimerization interface also presented conformations absent in the native interface. Interestingly, the association between ACE2 monomers is mediated by two different interfaces: one involving the PD domain and the other close to the cell membrane, i.e., the Neck domain. Besides peptides that were targeting individual interfaces, a number of them were also bridging both interfaces presenting both in helical and extended conformations (Figure 3D). Potentially these peptides could have an increased the affinity through a synergistic of each single interface.

### 2.4. Clusters of Designed Peptides Converging into a Common Conformation Can Be Used to Derive Sequence Profiles

**Figure 4:**
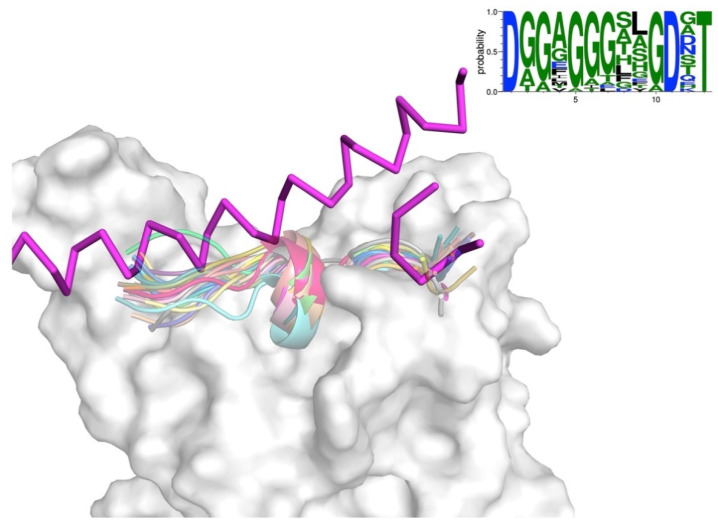
Structural ensemble of peptides targeting a common region of the target surface. RBD of SARS-CoV-2 S protein is shown in white and surface representation while the H1 helix of ACE2 is shown in magenta and ribbon. The different designed peptides are shown in different colours and translucent cartoon representation. A web logo [33] depicting the conservation is shown in the top right side.

The charting of the targeted surface is driven by the anchor residues and so there are cases where a specific peptide conformation(s) is (are) preferred in given region on the surface. In these cases, there is a convergence and so an ensemble of conformations can be identified. The important aspect is that this ensemble is not the result of perturbations to a starting conformation (e.g., backbone changes) but rather independent exploration during the structural modelling step. An example is presented in Figure 4. Some of the peptides targeting the central region of ACE2/RBD presented a common conformation composed of a helical turn flanked by two extended extremities (the main H1 helix is shown as a reference). It can be seen the extensive interactions with a central region of RBD, which although not actually engaged in the native interaction with ACE2, presents desirable feature such as cavities exploited by the designed peptides. As shown on the web logo, the sequences of these peptides presented certain conservation but also certain degree of diversity that could be exploited as a guide to design screening libraries.

### 2.5. Combining Structural Information from Different Complexes to Design Peptides

**Figure 5:**
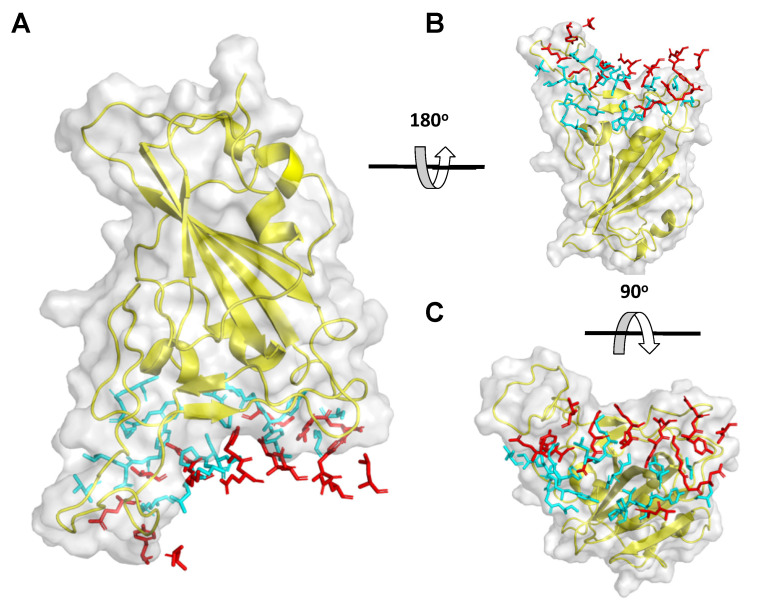
Overlap between ACE2 and mAb CB6 and RBD interfaces. RBD of SARS-CoV-2 S protein is shown in cartoon and surface representation (yellow) while the side chain of the interface residues of ACE2 and mAb CB6 are show in sticks representation in red and cyan respectively. Note that only the interface residues are shown to facilitate the visualization. Panels (**A**–**C**) show different orientations by applying the rotations shown.

Both ACE2 (Figure 1) and mAbs (Supplementary Figures S2 and S3) interact with the RBD although not sharing exactly the same interface (Figure 5). Given the flexible modelling strategy of PiPreD, it is possible to combine the structural information of multiple protein complexes that share a common partner [30]. On doing so, native elements can be incorporated from different complexes to increase the diversity of peptides and size of the targeted interface (i.e., larger surfaces). To that end, peptides were also modelled including the information of two different proteins complexes: RBD/ACE2 and RBD/mAb CB6 (Figure 5). The combined area of the interface was slightly larger than that of the individual complexes reflected in a larger number of anchor residues (62) available for modelling resulting in the largest pool of designed peptides (ca. 700 k).

### 2.6. PepI-Covid10 Database Repository: Access and Functionalities

An online repository to the designed peptides is freely accessible at the Pep*I*-Covid19 database (http://bioinsilico.org/pepicovid19/) (last accessed 27 October 2021). The repository is web interfaced allowing user to perform queries based on parameters such as the conformation of the peptides, size and predicted binding energy as per the Rosetta energy score [34]. Users can also query the information based on a number of interface statistics such the surface area of the interface between protein and peptides (in Ang^2^), the number of hydrogen bonds and unsatisfied hydrogen bonds (in case of buried donor or acceptors groups) at the interface, packing [35] or the peptide score. The query returns a list of the peptides that fulfils the conditions set on the query and can be filtered by sequence, and/or sorted by different parameters (e.g., size). Upon selecting a specific peptide, a page with specific information on the peptide is shown together with an applet allowing the three-dimensional visualization of the protein-peptide structural complex (Figure S5).

## 3. Materials and Methods

### 3.1. Protein Complexes

The crystallographic structures of a range of protein complexes were used. This includes the structure of the SARS-CoV-2 RBD bound with ACE2 (PDB code 6m0j) [29] and SARS-CoV-2 spike receptor-binding domain bound to full length ACE2 (PDB code 6m17) [4]. The structure of the RBD of SARS-CoV bound to ACE2 (PDB code 2ajf) [2] was also considered as contrasting exemplar given the fact that although showing overall structural similarity at fold level with the RBD of SARS-CoV-2, their interaction interfaces are different [32]. Furthermore, we included the structures of two monoclonal antibodies (PDB codes: 7c01 and 7k8m) [9,10] and a nanobody (PDB code 6yz5) bound to RBD.

### 3.2. Modeling and Design of Peptides

Peptides were modelled and designed using PiPreD and Rosetta [34] as previously described [30]. The modelling step relies on a library of iMotifs that are fitted in the interface to target using the so-called anchor residues. The parameters used in the modelling stage were: (i) a maximum distance between C-alpha iMotifs-anchors of 0.5 Ang., and (ii) a root mean square deviation value smaller than 1.0 Ang. upon structural superposition iMotifs-anchors. Once the iMotifs were structurally fitted and prior to the designing stage, peptides with low interface packing and mimicking less than 4 anchor residues were discarded. The design of peptides was done using the backrub application [36] within the Rosetta suite [34]. The backrub motions allow the limited movement of the main chain of the peptide while optimizing the interactions with the interface while allowing the design of any position not structurally aligned with anchor residues, where residue type is preserved although rotameric changes were allowed. The residues on the interface belonging to the protein were also allowed to repack. Once the designing stage was completed, the FlexPepDock application [37] in “*score mode only*” was used to derived several scores (i.e., interface score or peptide score) and other measures such as the buried surface area, number of hydrogen bonds and other metrics.

### 3.3. Database Design, Implementation and Interfacing

Information about the designed peptides is stored in an SQLite3 database. The web interface accessible for the user is generated with the Flask Python Microframework. The structure of the complex protein-peptides is rendered with PV, a WebGL-based JavaScript API (https://biasmv.github.io/pv/) (last accessed 30 September 2021). The repository is available at http://bioinsilico.org/pepicovid19 (last accessed 30 September 2021).

## 4. Conclusions

Here we present a repository of computationally designed peptides to interfere in the early stages of invasion process by SARS-CoV-2. These peptides present a large structural diversity, targeting both the RBD/ACE2, as well as the extracellular ACE2/ACE2 interfaces. We include peptides to target both SARS-CoV and SARS-CoV-2 RBD that although sharing an overall structural similarity present however distinct interaction surfaces reflected in the low overlap of peptides sequences. Structurally equivalent peptides targeting common regions of protein surfaces can be used to derive sequence profiles that be used to guide library design or high-throughput peptides synthesis arrays. Moreover, the flexibility of the modelling and designing method allowed to combine information for different protein complexes to increase the diversity of the peptides.

Although we are aware of the limitations of computational predictions, we believe that the information presented here is a significant and valuable asset to current efforts devoted to find novel therapeutic agents and strategies to fight SARS-CoV-2. Indeed, the information of protein sequences of potential peptide inhibitors can be used as guidance for experimental efforts and structural model of peptides can represents the basis for further computational work, e.g., molecular dynamic simulations to assess the strength of binding. We have established a freely accessible online repository, Pep*I*-Covid19 database, to allow the access, search and a convenient retrieval of information including the interactive visualization of the 3D structure of protein-peptide complexes. Finally, and within the limits of our finite resources, the repository will be updated as structural information becomes available on protein complexes related to SARS-CoV-2.

## Figures and Tables

**Table 1:** List of protein complexes considered and interface features.

Protein Complex	PDB Code	Interface Area (Ang^2^)	Number of Anchor Residues
SARS-CoV-2 RBD/ACE2	6m0j 6m17	2032.01	30
ACE2/ACE2	6m17	2721.41	39
SARS-CoV RBD/ACE2	2ajf	1917.98	30
SARS-CoV-2 RBD/mAb CB6	7c01	2123.29	32
SARS-CoV-2 RBD/mAb c102	7k8m	2028.28	38
SARS-CoV-2 RBD/Nanobody H11D4	6yz5	1126.42	18

## Data Availability

The data presented in this study is included in the article and its Supplementary Materials. The entire repository of the designed peptides is available through the web server listed (http://bioinsilico.org/pepicovid19) (last accessed 30 September 2021). Requests of different nature can also be to the corresponding authors on a reasonable basis.

## Supplementary Materials

The following are available online at https://www.mdpi.com/article/10.3390/ijms222111627/s1.
