# Supplementary material for: A Collection of Designed Peptides to Target SARS-CoV-2 Spike RBD—ACE2 Interaction
Source: Int J Mol Sci. 2021 Oct 27;22(21):11627. doi: 10.3390/ijms222111627 (PMC8584250; doi:10.3390/ijms222111627)
Supplement: Supplementary file 1 [file ijms-22-11627-s001.zip › ijms-1423835-supplementary.pdf]

SUPPLEMENTARY FIGURES

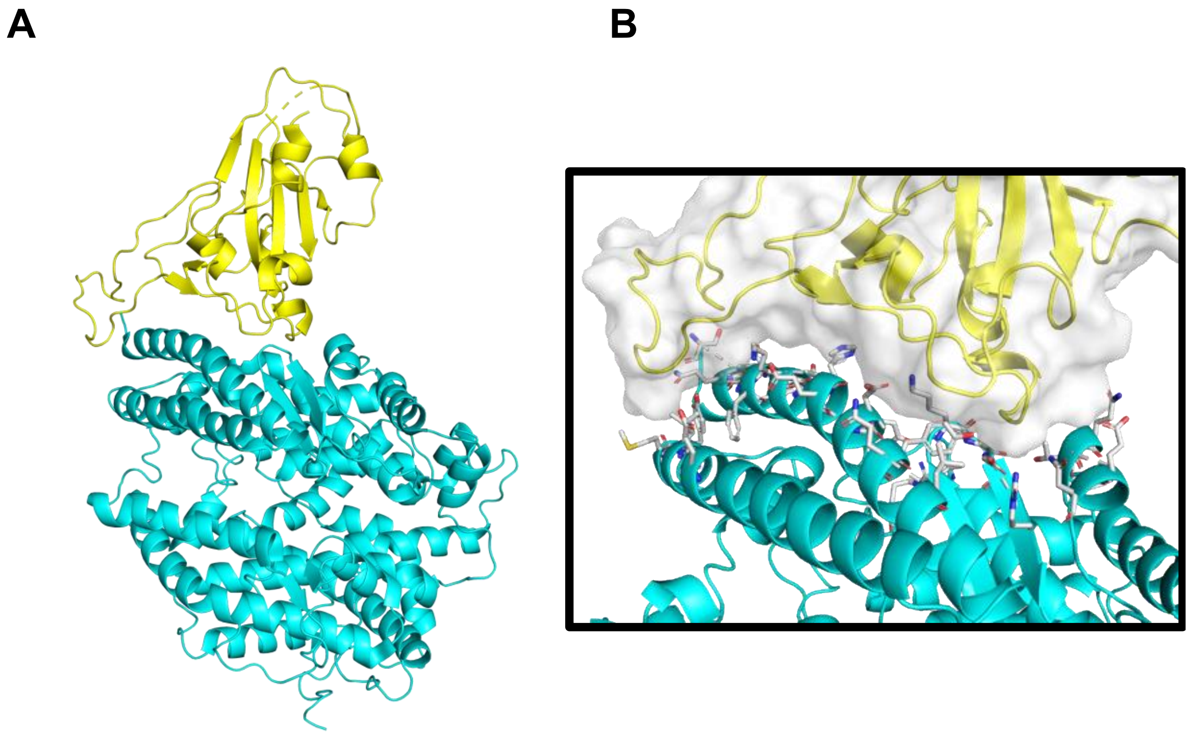

Supplementary Figure S1. Interface SARS-CoV RBD / ACE2. (A) Cartoon representation of human ACE2 (cyan) and RBD (yellow). (B) Interface targeted by peptides. Enlarged view of the interface between ACE2 (cyan, cartoon; interface residues in sticks representation) and RBD (yellow, surface).

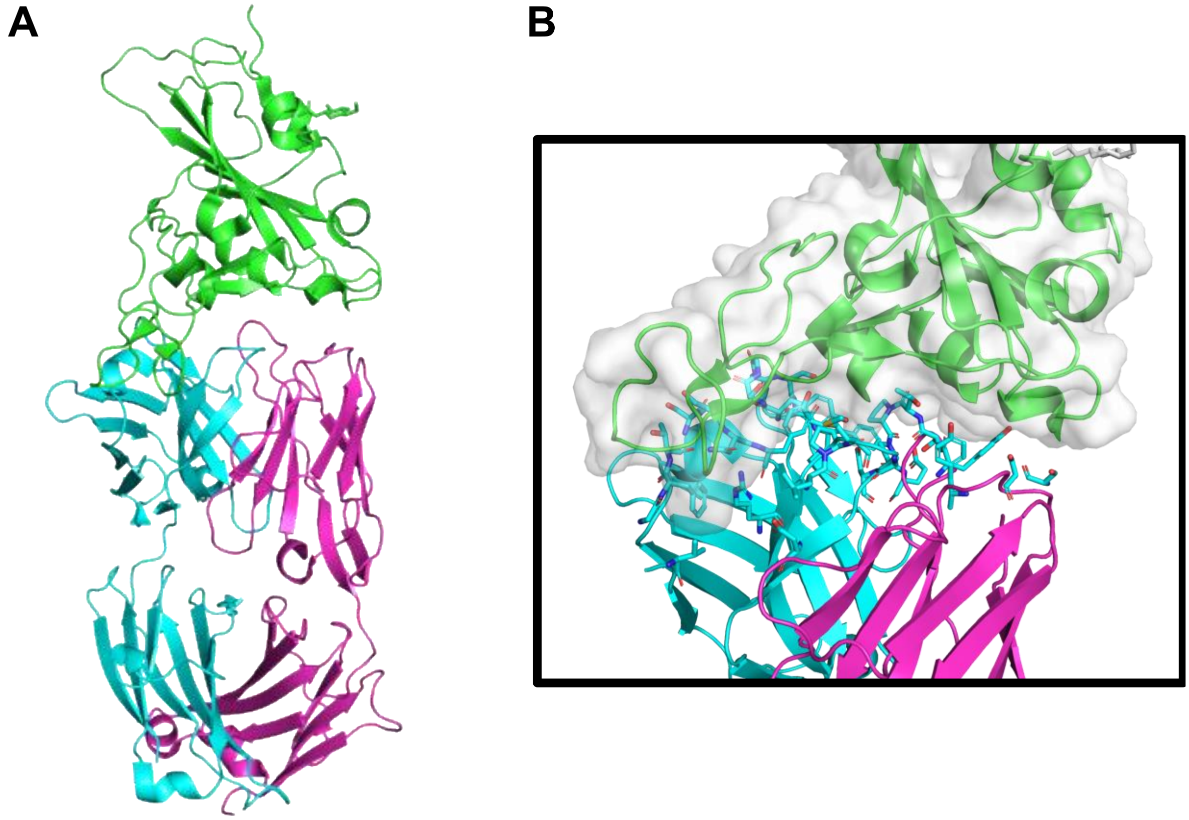

Supplementary Figure S2. Interface SARS-CoV-2 RBD / mAb CB6. (A) Cartoon representation of mAb CB6 (cyan and magenta) and RBD (green). (B) Interface targeted by peptides. Enlarged view of the interface between mAb CB6 (cyan and magenta, cartoon; interface residues in sticks representation) and RBD (green, surface).

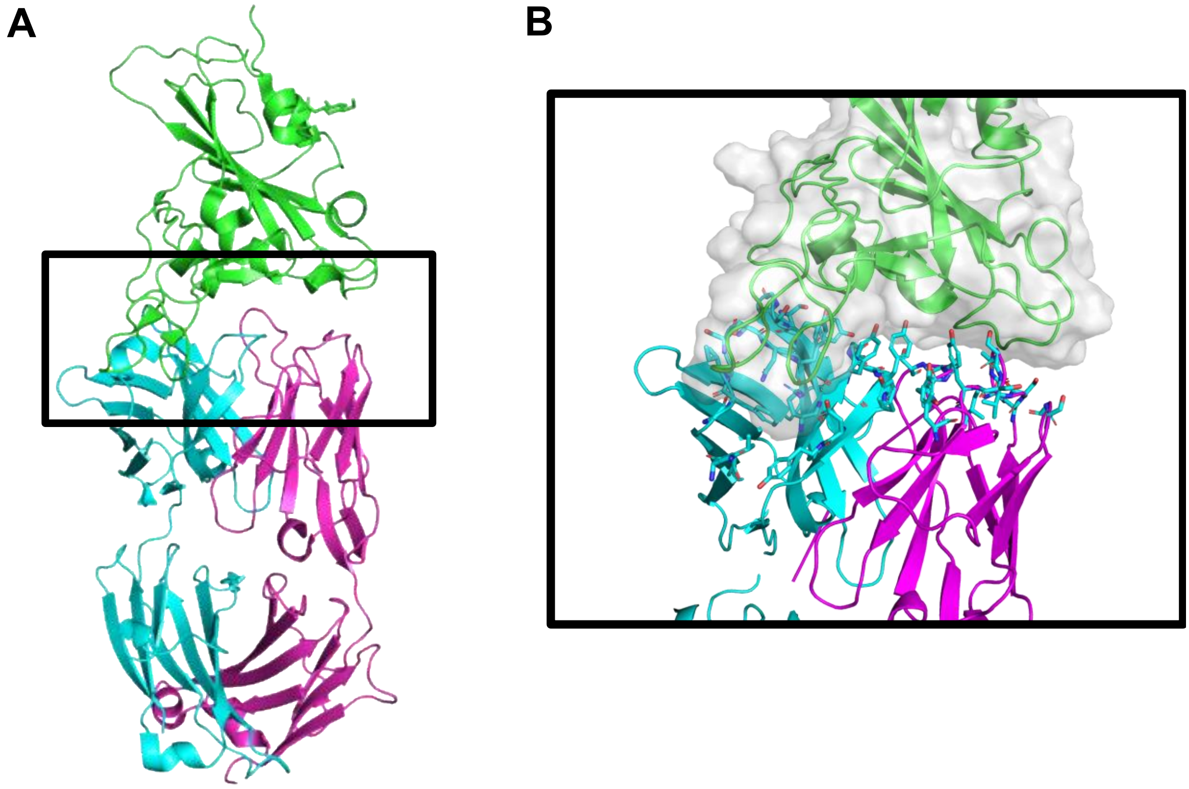

Supplementary Figure S3. Interface SARS-CoV-2 RBD / mAb C102. (A) Cartoon representation of mAb C102 (cyan and magenta) and RBD (green). (B) Interface targeted by peptides. Enlarged view of the interface between mAb C102 (cyan and magenta, cartoon; interface residues in sticks representation) and RBD (green, surface).

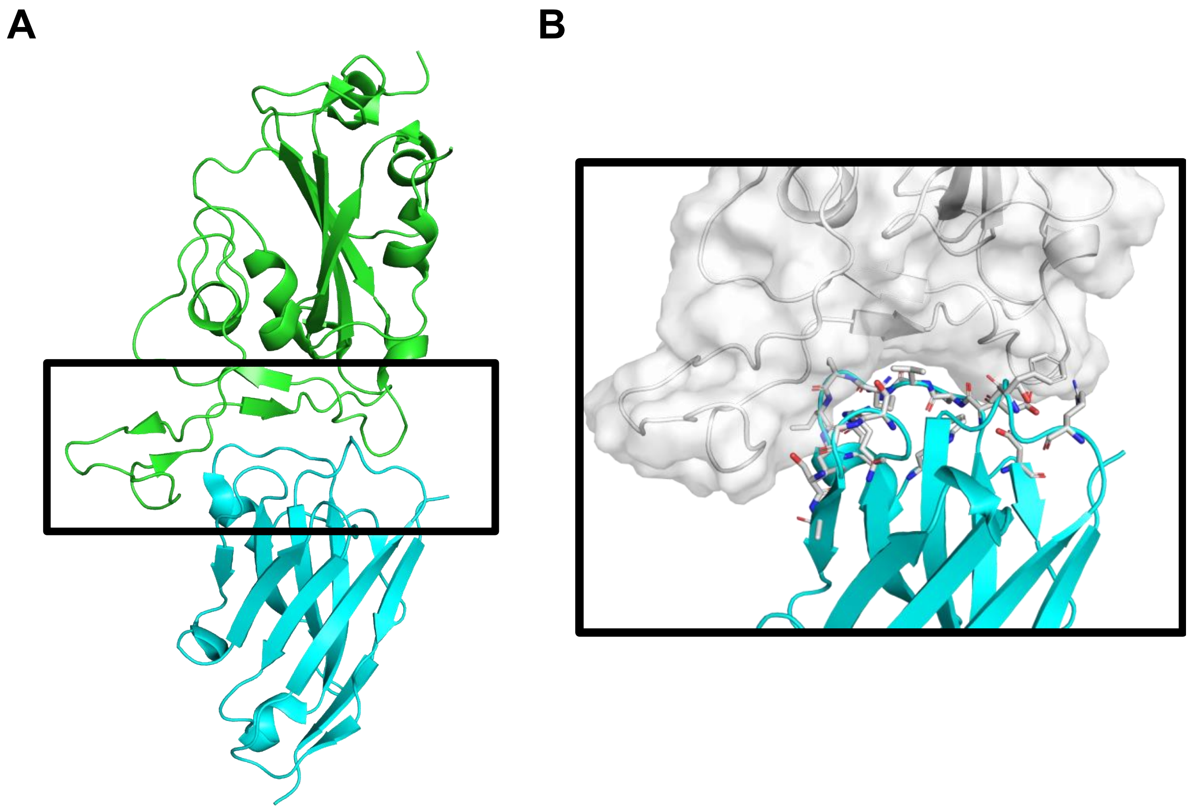

Supplementary Figure S4. Interface SARS-CoV-2 RBD / nanobody H11 D4. (A) Cartoon representation of nanobody H11 D4 (cyan) and RBD (green). (B) Interface targeted by peptides. Enlarged view of the interface between nanobody H11 D4 (cyan and cartoon; interface residues in sticks representation) and RBD (gray, surface).

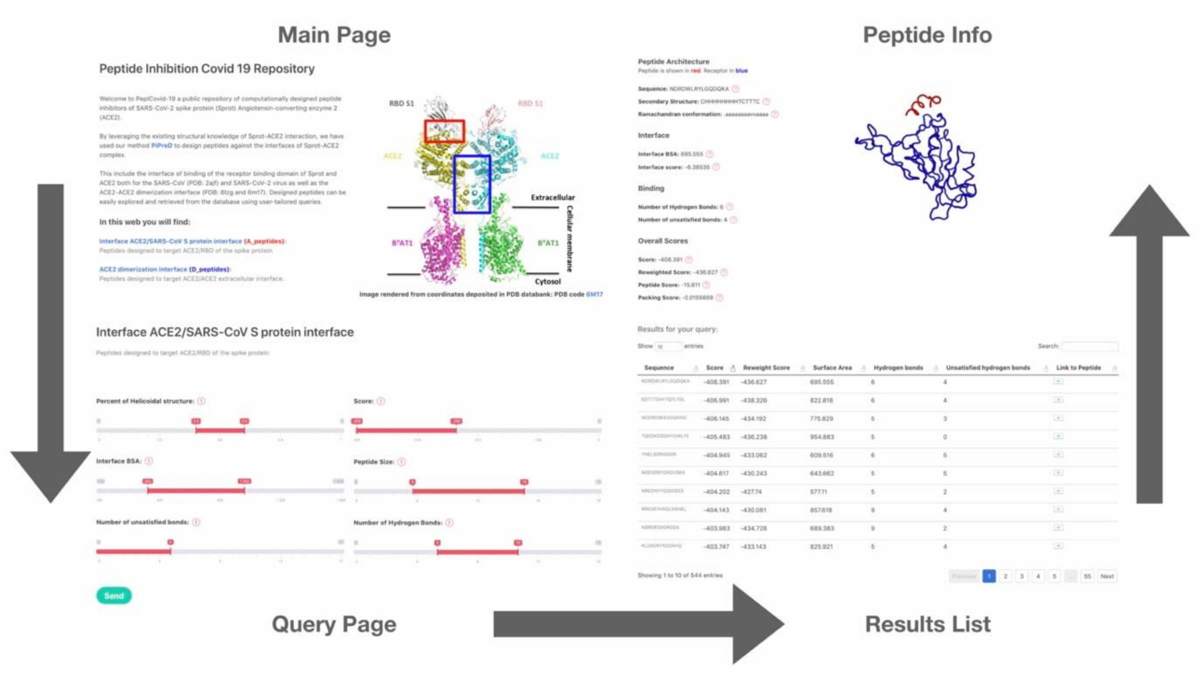

Supplementary Figure S5. Pep*I*-Covid19 database web interface. From top to bottom and left to right the different web-pages shown in the repository that allow users to query, search and filter results as well as visualize the three-dimensional structure of protein complex interactively.
